# Allgemein- und viszeralchirurgische Oberarztkonsile für andere medizinische Disziplinen über 10 Jahre an einem tertiären Zentrum – ist eine schnelle, zeitaufwendige Abarbeitung notwendig? Klinisches Befund‑, Diagnose- und therapeutisches Entscheidungsspektrum

**DOI:** 10.1007/s00104-023-01855-4

**Published:** 2023-03-29

**Authors:** C. Schildberg, S. Kropf, A. Perrakis, R. S. Croner, F. Meyer

**Affiliations:** 1 Klinik für Allgemein und Viszeralchirurgie, Universitätsklinikum der MHB im Verbund Brandenburg an der Havel, Hochstraße 29, 14770 Brandenburg an der Havel, Deutschland; 2 Institut für Biometrie und Medizinische Informatik, Universitätsklinikum Magdeburg A.ö.R., Magdeburg, Deutschland; 3 Klinik für Allgemein‑, Viszeral‑, Gefäß- und Transplantationschirurgie, Universitätsklinikum Magdeburg A.ö.R., Magdeburg, Deutschland

**Keywords:** Observationsstudie, Fachdisziplinen, Diagnoseprofil, Geschlechtsunterschied, Altersunterschied, Observational study, Specialist disciplines, Diagnosis profile, Sex difference, Age difference

## Abstract

**Hintergrund:**

Die Herausforderungen einer adäquaten, effizienten und rationellen medizinischen Versorgung und Betreuung von Patienten stehen immer im Zusammenhang mit der interprofessionellen Tätigkeit mehrerer Fachdisziplinen.

**Ziel:**

Die Breite variabler Diagnosen und des Profils der chirurgischen Entscheidungsfindung mit weiterführenden chirurgischen Maßnahmen im Rahmen des allgemein- und viszeralchirurgischen Oberarztkonsils für medizinische Nachbardisziplinen wurde über einen definierten Beobachtungszeitraum an einer repräsentativen Patientenklientel analysiert.

**Patienten und Methode:**

Über 10 Jahre (01.10.2006 bis 30.09.2016) wurden alle konsekutiven Patienten (*n* = 549 Fälle) im Rahmen einer klinisch-systematischen, prospektiven unizentrischen Observationsstudie an einem tertiären Zentrum in einer computerbasierten Patientendatei erfasst und hinsichtlich klinischem Befund‑, Diagnose- und therapeutischem Entscheidungsspektrum und ihrer signifikanten Einflussfaktoren sowie Geschlechts- und Altersunterschied als auch hinsichtlich zeitabhängiger Entwicklungstrends mittels χ^2^-Test und *U*-Test analysiert.

**Ergebnisse (Eckpunkte):**

Die dominierende Fachdisziplin der Konsilabforderung war die Kardiologie (19,9 %), gefolgt von anderen chirurgischen Fächern (11,8 %) und der Gastroenterologie (11,3 %). Das Diagnoseprofil wurde von Wundheilungsstörungen (7,1 %) und akutem Abdomen (7,1 %) bestimmt. Bei 11,7 % der Patienten wurde die unmittelbare Operationsindikation gestellt und bei 12,9 % wurde die Operation elektiv empfohlen. Die Übereinstimmungsrate von Verdachts- und definitiver Diagnose lag bei lediglich 58,4 %.

**Schlussfolgerung:**

Die chirurgische Konsiltätigkeit ist ein wichtiges Standbein für die suffiziente und vor allem zeitgerechte Klärung chirurgisch relevanter Fragestellungen in fast jeder medizinischen Einrichtung, so vor allem auch in einem Zentrum. Sie dient i) der chirurgischen Qualitätssicherung bei der klinischen Mitbetreuung von Patienten mit interdisziplinärem, hier chirurgischem Versorgungsbedarf in der täglichen allgemein-/viszeralchirurgischen Praxis im Rahmen der klinischen Versorgungsforschung; ii) dem Klinikmarketing sowie monetären Aspekten im Sinne von Patientenrekrutierung (und) iii) nicht zuletzt der Notfallversorgung von Patienten. Aufgrund des hohen Anteils von 12 % folgenden Notfalloperationen nach gestellter allgemein-/viszeralchirurgischer Konsilanforderung sind diese zeitnah in der Dienstzeit abzuarbeiten.

## Abstract (en)

**Background:**

The challenges of an adequate, efficient and rational medical treatment and care of patients are always associated with an interprofessional activity of several specialist disciplines.

**Aim:**

The spectrum of variable diagnoses and the profile of surgical decision-making with further surgical measures within the framework of senior physician consultation in general and visceral surgery for neighboring medical disciplines were analyzed on a representative patient cohort over a defined observational time period.

**Patients and methods:**

All consecutive patients (*n* = 549 cases) were documented as part of a clinical systematic prospective single center observational study at a tertiary center using a computer-based patient registry over 10 years (1 October 2006–30 September 2016). The data were analyzed with respect to the spectrum of clinical findings, diagnoses, treatment decisions and the influencing factors as well as gender and age differences and time-dependent developmental trends using χ^2^-tests and U‑tests.

**Results (key points):**

The predominant discipline for requests for surgical consultation was cardiology (19.9%) followed by surgical disciplines (11.8%) and gastroenterology (11.3%). Disorders of wound healing (7.1%) and acute abdomen (7.1%) were predominant in the diagnostic profile. In 11.7% of the patients the indications for immediate surgery were derived, whereas in 12.9% elective surgery was recommended. The conformity rate of suspected and definitive diagnoses was only 58.4%.

**Conclusion:**

The surgical consultation work is an important mainstay of a sufficient and especially timely clarification of surgically relevant questions in nearly all medical institutions and especially in a center. This serves i) the quality assurance of surgery in the clinical care of patients with need of additional interdisciplinary needs for surgical treatment in the daily practice of general and abdominal surgery in research on clinical care, ii) clinical marketing and monetary aspects in the sense of patient recruitment and iii) last but not least to provide emergency care of patients. Due to the high proportion of 12% of subsequent emergency operations, which were derived from requests for general and visceral surgical consultations, such requests must be processed promptly during working hours.

## Hintergrund

Die Herausforderungen einer adäquaten, effizienten und rationellen medizinischen Versorgung und Betreuung von Patienten stehen immer im Zusammenhang mit der interprofessionellen Tätigkeit mehrerer Fachdisziplinen, z. B. realisiert durch primäre Konsilanforderungen, gerade an tertiären Zentren oder Häusern der Maximalversorgung mit anspruchsvollen spezifischen Fallkonsultationen. Dabei wird diese nicht selten zeitaufwendige Tätigkeit gelegentlich subjektiv als lästig empfunden, da sich vermeintliche Notfälle als teils nicht so gravierend herausstellen.

Das Ziel der Untersuchung war es, die Breite variabler Diagnosen und das Profil der chirurgischen Entscheidungsfindung mit weiterführenden chirurgischen Maßnahmen im Rahmen des allgemein- und viszeralchirurgischen Oberarztkonsils für medizinische Nachbardisziplinen über einen definierten Beobachtungszeitraum an einer repräsentativen Patientenklientel in einem klinischen Zentrum der Maximalversorgung zu untersuchen. Dabei wurde besonderes Augenmerk auf die inhaltliche Übereinstimmung mit der Anforderung und dem vorgefundenen Krankheitsbild gelegt. Zudem sollte der Anteil an Notfalloperationen, die einen deutlichen Zeitaufwand für die Diensttätigkeit beinhalten, herausgearbeitet werden. In diesem Zusammenhang sollte nicht zuletzt auch geklärt werden, ob eine schnelle Abarbeitung überhaupt indiziert ist.

## Patienten und Methode

Über 10 Jahre wurden alle konsekutiven Patienten einer singulären allgemein- und viszeralchirurgischen Oberarztkonsiltätigkeit (ein ärztlicher Kollege) für andere medizinische Disziplinen im Rahmen einer klinisch-systematischen, prospektiven unizentrischen Observationsstudie an einem tertiären Zentrum

- sowohl im elektiven als auch Notfallsetting,
- hinsichtlich Geschlechtsunterschied,
- im Altersvergleich (< 65 vs. > 65 Jahre) und
- in Gegenüberstellung von 2,5-Jahres-Teilzeiträumen für eine mögliche Trendbeurteilung

in einer computerbasierten Patientendatei erfasst.

Insbesondere wurden das klinische Befund‑, Diagnose- und therapeutische Entscheidungsspektrum dokumentiert wie z. B.:

- Übereinstimmungsrate von Verdachts- und definitiver Diagnose,
- Rate der gestellten Operationsindikation u. a.

Einflussfaktoren auf die chirurgische Entscheidungsfindung, den Geschlechts- oder Altersunterschied wurden, wie jeweils angezeigt, mittels χ^2^-Test bzw. *U*-Test analysiert. Ein *p*-Wert von < 0,05 wurde als statistisch signifikant eingestuft.

Die Studie wurde entsprechend den Bestimmungen der Deklaration von Helsinki für Biomedizinische Forschung von 1964 und ihren weiteren aktualisierten bzw. erweiterten Durchführungsbestimmungen als auch entsprechend den aktuellen Vorgaben der hiesigen institutionellen Ethikkommission sowie den Regeln der „good clinical and research practice“ durchgeführt.

## Ergebnisse

**Figure 1:**
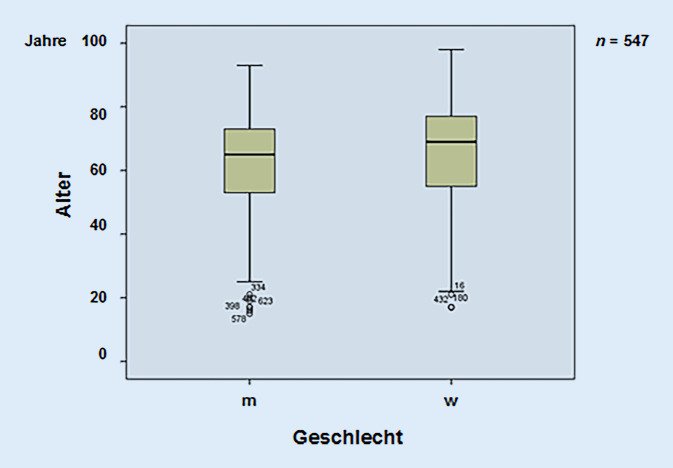

Vom 01.10.2006 bis 30.09.2016 wurden insgesamt 549 Fälle dokumentiert (Geschlechtsverhältnis: männlich:weiblich = 321:228 [1,41: 1]; Altersmedian: 63 [Streubreite: 15–98] Jahre; Abb. 1 und 2). Der vierte 2,5-Jahres-Zeitraum von 04/2014 bis 09/2016 wies anteilmäßig das höchste Fallaufkommen mit 302 Patienten*innen auf, was insbesondere auf die verantwortliche Oberarzttätigkeit im Ambulanzbereich zurückzuführen war (ähnliches Geschlechterverhältnis: männlich:weiblich  = 185:117 [1,58: 1]; Durchschnittsalter: 62 [Streubreite: 15–98] Jahre) ohne wesentliche tendenzielle Änderungen in der Patientendemografie (Tab. 1, VIII).

**Figure 2:**
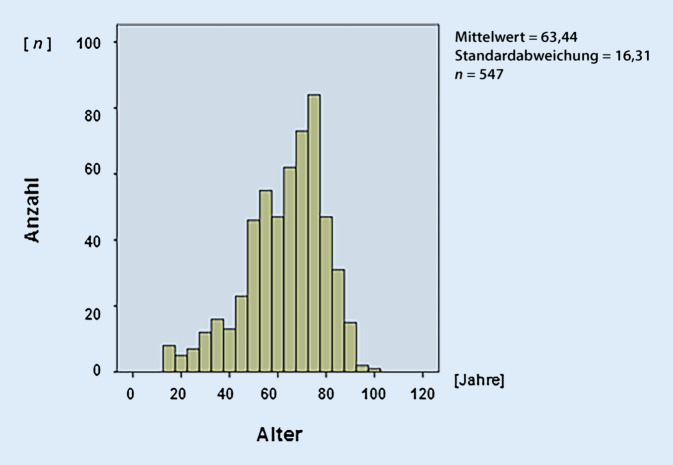

**Table 1:** 

*I) Demografie*				
**Auswertparameter**	**Anzahl [*n*]**		**Relativer Anteil [%]**	
*Patienten (männlich)*	321		58,5	
(bis 65 Jahre)	(164)		(61,4)	
(ab 66 Jahre)	(157)		(56,1)	
*Patienten (weiblich)*	228		41,5	
(bis 65 Jahre)	(103)		(38,6)	
(ab 66 Jahre)	(123)		(43,9)	
*Total*	*549*		*100,0*	
**Altersmedian (Streubreite)**	**63 (15–98) Jahre**			
Patienten (10/2006–03/2008)	45		8,2	
Patienten (04/2008–09/2010)	120		21,9	
Patienten (10/2010–03/2014)	82		14,9	
Patienten (04/2014–09/2016)	302		55,0	
*Total*	*549*		*100,0*	
***II) Studiencharakteristika***				
Design (Ziel)	Klinisch-systematische, prospektive unizentrische Observationsstudie (zur Reflexion des chirurgischen Alltags als Beitrag zur chirurgischen Qualitätssicherung im Rahmen der klinischen Versorgungsforschung [computerbasierte Patientendatei] an einem tertiären Zentrum)			
Dauer	10 Jahre (10/2006–09/2016)			
Unterteilungen/Gegenüberstellungen	Klinisches Befund‑, Diagnose- und therapeutisches Entscheidungsspektrum			
	Elektive vs. Notfallkonsile			
	Frauen vs. Männern			
	Patienten ≤ 65 vs. > 65 Jahre			
	2,5-Jahres-Zeiträume			
***III) Profil konsilanfordernder Disziplinen (n*** _***total***_ ***=*** ***549)***^***a***^				
**Nachbardisziplinen**	**Anzahl [*n*]**		**Relativer Anteil [%]**	
Kardiologie	109		19,9	
Nichtallgemein‑/Viszeralchirurgie	65		11,8	
Gastroenterologie	62		11,3	
Notaufnahme	55		10,0	
Nichtchirurgische Intensivstationsbereiche	48		8,7	
Neurologie	47		8,6	
***IV) Spektrum der Konsultationsgründe***^***a***^				
**Auswertparameter**^**b**^	**Anzahl [*n*]**		**Relativer Anteil [%]**	
Mitbeurteilung	383/549		69,8	
Therapieempfehlung	79/549		14,4	
Verlaufskontrolle	88/549		16,0	
Wiedervorstellung	13/549		2,4	
Operationsindikation	134/549		24,4	
Postoperative Wiedervorstellung	38/549		6,9	
***V) Spektrum führender Diagnosen***^***a***^				
**Diagnosen**^**d**^	**Anzahl [*n***] **suspekt**	**Relativer Anteil [%]** **suspekt**	**Anzahl [*n***]** definitiv**	**Relativer Anteil [%]** **definitiv**
Wundheilungsstörung	33	6,0	39	7,1
Platzbauch	0	0,0	1	0,2
Unklares Abdomen	23	4,2	11	2,0
Akutes Abdomen	65	11,8	39	7,1
Hernie	29	5,3	20	3,6
Perforation	7	1,3	5	0,9
Abszess/Phlegmone	34	6,2	22	4,0
Hämatom	18	3,3	26	4,7
Blutung	38	6,9	26	4,7
Ileus	16	2,9	5	0,9
Ischämie	13	2,4	13	2,4
Cholezystitis	18	3,3	14	2,6
Cholezystolithiasis	13	2,4	9	1,6
Karzinom	17	3,1	8	1,5
Sonstiger Tumor	14	2,6	13	2,4
Pneumonie	9	1,6	10	1,8
Sonstige^c^	330	60,1	309	56,3
***VI) Empfohlene medizinische Maßnahmen (n*** _***total***_ ***=*** ***549)***^***a***^				
**Medizinische Maßnahmen**	**Anzahl [*n*]**		**Relativer Anteil [%]**	
Bildgebende Diagnostik	223/549		40,6	
Labor	207/549		37,7	
Elektrokardiographie/Elektroenzephalographie	13/549		2,4	
Darmrohr	35/549		6,4	
Sphinkterdehnung (anal)	38/549		6,9	
Weiteres Fachkonsil	235/549		42,8	
Operationsempfehlung (elektiv)	71/549		12,9	
*Operationsindikation*	*64/549*		*11,7*	
Davon aus Notfällen	6/64		1,1	
Patientenübernahme	71/549		12,9	
***VII) Übereinstimmung im Vergleich der suspekten und definitiven Diagnosen (n ***_***auswertbar***_ ***=*** ***339; n ***_***total***_ ***=*** ***549)***				
**Diagnosen**	**Anzahl [*n*]**		**Relativer Anteil [%]**	
*Total*	*549*		*100,0*	
Nicht Auswertbar	210		38,3	
Auswertbar *Ja* *Nein*	339 *198* *141*		61,7 *58,4* *41,6*	
***VIII) Zeitraumvergleich (4*** ***×*** ***2,5 Jahre)***^***a***^***/***^***e***^				
–	**Zeitraum 1 [*n*** **=** **45 Pat.]** **Relativer Anteil [%]**	**Zeitraum 2 [*n*** **=** **120 Pat.]** **Relativer Anteil [%]**	**Zeitraum 3 [*n*** **=** **82 Pat.]** **Relativer Anteil [%]**	**Zeitraum 4 [*n*** **=** **302 Pat.]** **Relativer Anteil [%]**
*Zu I) Demografie*				
Patienten (männlich)	48,8	61,7	52,4	61,3
Patienten (weiblich)	51,2	38,3	47,6	38,7
*Zu IV) Konsultationsgrund*				
Mitbeurteilung	46,7	54,2	73,2	90,7
Operationsindikation	24,4	18,3	14,6	32,1
*Zu V) Definitive Diagnosen*				
Wundheilungsstörung	4,4	9,2	7,3	6,3
Akutes Abdomen	20,0	8,3	8,5	13,6
Blutung	6,7	8,3	7,3	6,3
*Zu VI) Medizinische Maßnahmen*				
Bildgebende Diagnostik	53,3	37,5	37,8	49,0
Labor	51,1	36,7	39,0	44,7
Weiteres Fachkonsil	35,6	50,0	59,8	46,3
Operationsempfehlung (elektiv)	8,9	8,3	8,5	18,2
Operationsindikation	22,2	10,0	6,1	13,6

^a^Mehrfachnennung bei einzelnen Patienten möglich

^b^Reihenfolge nach Wertigkeit im klinischen Vorgehen

^C^Unter Sonstige zählen: u. a. Pankreatitis, periphere arterielle Verschlusskrankheit, Thrombose, Narbenbruch

^d^Verdachtsdiagnosen bei Konsiliaranforderung „(suspekt)“ vs. chirurgischerseits festgestellte Diagnosen, die für die weitere befundspezifische Versorgung zugrunde gelegt wurde „(definitiv)“

^e^Jeweils nur Kategorien mit größten prozentualen Anteilen der Teiltabellen I, IV, V und VI aufgeführt

*„Pat.“* Patient

Die dominierende Fachdisziplin der Konsilabforderung war die Kardiologie (*n* = 109/549; 19,9 %), gefolgt von nichtallgemein- und viszeralchirurgischen Teildisziplinen (*n* = 65/549; 11,8 %) und der Gastroenterologie (*n* = 62/549; 11,3 %). Wie bereits oben erwähnt wurde die Konsiltätigkeit durch einen oberärztlichen Kollegen durchgeführt. Bei den Kollegen*innen, die das Konsil angefordert haben, handelte es sich um alle Ausbildungsstätten, vornehmlich jedoch Assistenten*innen.

Das Spektrum der Konsultationsgründe umfasste (Reihenfolge nach Wertigkeit im klinischen Vorgehen):

- eher klinisch-beurteilend geprägt: „Mitbeurteilung“ zu 69,8 % (*n* = /549), „Therapieempfehlung“ 14,4 % (*n* = 79/549), „Verlaufskontrollen“ 16 % (*n* = 88/549), „Wiedervorstellungen“ 2,4 % (*n* = 13/549),
- operationsassoziiert: Frage der Operationsindikation 24,4 % (*n* = 134/549) und postoperative Wiedervorstellungen 6,9 % (*n* = 38/549).

Das Diagnoseprofil war breit aufgestellt und wurde dominierend von Wundheilungsstörungen (*n* = 39/549; 7,1 %) und akutem Abdomen (*n* = 39/549; 7,1 %) bestimmt; eher weniger häufig waren Platzbauch (*n* = 1/549; 0,2 %), Perforation (*n* = 5/549; 0,9 %) und manifester Ileus (*n* = 5/549; 0,9 %) (Tab. 1, V).

Bei 11,7 % (*n* = 64/549) der Patienten wurde die unmittelbare Operationsindikation gestellt (davon kamen aus der hiesigen „Zentralen Notaufnahme“ lediglich mit 1,1 % [*n* = 6/64] als Notfall!) und in 12,9 % (*n* = 71/549) wurde die Operation elektiv empfohlen. Weitere abgeleitete Maßnahmen beinhalteten: u. a. „Labor“ 37,7 % (*n* = 207/549), „bildgebende Diagnostik“ 40,6 % (*n* = 223/549) und „Empfehlung zu einem weiteren Fachkonsil“ 42,8 % (*n* = 235/549). Bei der „bildgebenden Diagnostik“ handelt es sich bei einer unklaren klinischen Situation – wie im hausinternen Prozedere festgelegt – um computertomographische (CT‑)Untersuchungen. Die allgemein- und viszeralchirurgisch geprägten Fälle veranlassten auch zu Maßnahmen wie Darmrohr und anale Sphinkterdehnung in 6,4 % (*n* = 35/549) bzw. 6,9 % (*n* = 38/549) der Fälle (Tab. 1, VI).

Bei 61,7 % (*n* = 339/549) der registrierten Fälle war die Übereinstimmungsrate von Verdachts- und definitiver Diagnose auswertbar, die bei lediglich 58,4 % (*n* = 198/339) lag (Tab. 1, VII).

Im Geschlechtervergleich wurde, bezüglich des definitiven Diagnosespektrums bei weniger Frauen (Tab. 2; *n* = 228) als Männer (*n* = 321), häufiger eine Ischämie (*n* = 9/13) in der weiblichen Patientenklientel diagnostiziert (*p* = 0,048).

**Table 2:** 

Diagnosen (definitiv)		Geschlecht (m) *n* = 321		Geschlecht (w) *n* = 228			Altersgruppe (bis 65 Jahre) *n* = 267		Altersgruppe (ab 66 Jahre) *n* *=* *280*		
	Anzahl [*n*]	Anzahl [*n*]	Relativer Anteil [%]	Anzahl [*n*]	Relativer Anteil [%]	*p*-Wert	Anzahl [*n*]	Relativer Anteil [%]	Anzahl [*n*]	Relativer Anteil [%]	*p*-Wert
Wundheilungsstörung	39	20	6,2	19	8,3	0,400	22	8,2	17	6,1	0,406
Platzbauch	1	0	0,0	1	0,4	0,415	1	0,4	0	0,0	0,488
Unklares Abdomen	11	6	1,9	5	2,2	1,000	3	1,1	8	2,9	0,223
Akutes Abdomen	39	21	6,5	18	7,9	0,614	19	7,1	20	7,1	1,000
Hernie	20	14	4,4	6	2,6	0,359	8	3,0	12	4,3	0,498
Perforation	5	4	1,2	1	0,4	0,408	2	0,7	3	1,1	1,000
Abszess/Phlegmone	22	13	4,0	9	3,9	1,000	12	4,5	10	3,6	0,666
Hämatom	26	11	3,4	15	6,6	0,103	13	4,9	13	4,6	1,000
Blutung	26	14	4,4	12	5,3	0,685	7	2,6	18	6,4	**0,040**
Ileus	5	3	0,9	2	0,9	1,000	2	0,7	3	1,1	1,000
Ischämie	13	4	1,2	9	3,9	**0,048**	4	1,5	9	3,2	0,263
Cholezystitis	14	11	3,4	3	1,3	0,170	7	2,6	7	2,5	1,000
Cholezystolithiasis	9	7	2,2	2	0,9	0,317	5	1,9	4	1,4	0,747
Karzinom	8	3	0,9	5	2,2	0,286	2	0,7	6	2,1	0,287
Sonstiger Tumor	13	7	2,2	6	2,6	0,780	3	1,1	10	3,6	0,090
Pneumonie	10	5	1,6	5	2,2	0,748	1	0,4	9	3,2	**0,021**
Sonstige^b^	309	185	57,6	124	54,4	0,485	152	56,9	156	55,7	0,796

^a^Mehrfachnennung bei einzelnen Patienten möglich

^b^Unter Sonstige zählen: u. a. Pankreatitis, periphere arterielle Verschlusskrankheit, Thrombose, Narbenbruch

Im Vergleich der Altersgruppen bestand bei Patienten ab 66 Jahre (Tab. 2; *n* *=* 280) ein höheres Risiko, eine manifeste Pneumonie (*n* *=* 9/10) aufzuweisen (*p* *=* 0,021).

Im Spektrum der chirurgisch-konsiliarisch vorgestellten Verdachtsdiagnosen war weder ein Unterschied hinsichtlich des Geschlechts noch der verglichenen Altersgruppen zu verzeichnen (Tab. 3).

**Table 3:** 

Diagnosen (suspekt)		Geschlecht (m) *n* = 321		Geschlecht (w) *n* = 228			Altersgruppe (bis 65 Jahre) *n* = 267		Altersgruppe (ab 66 Jahre) *n* *=* *280*		
	Anzahl [*n*]	Anzahl [*n*]	Relativer Anteil [%]	Anzahl [*n*]	Relativer Anteil [%]	*p*-Wert	Anzahl [*n*]	Relativer Anteil [%]	Anzahl [*n*]	Relativer Anteil [%]	*p*-Wert
Wundheilungsstörung	33	19	5,9	14	6,1	1,000	15	5,6	18	6,4	0,723
Platzbauch	0	0	0,0	0	0,0	–	0	0,0	0	0,0	–
Unklares Abdomen	23	12	3,8	11	4,8	0,666	13	4,9	10	3,6	0,525
Akutes Abdomen	65	36	11,2	29	12,7	0,595	27	10,1	38	13,6	0,235
Hernie	29	20	6,2	9	3,9	0,254	14	5,2	15	5,4	1,000
Perforation	7	2	0,6	5	2,2	0,133	1	0,4	6	2,1	0,123
Abszess/Phlegmone	34	19	5,9	15	6,6	0,858	18	6,7	16	5,7	0,724
Hämatom	18	10	3,1	8	3,5	0,812	10	3,7	8	2,9	0,636
Blutung	38	21	6,5	17	7,5	0,734	17	6,4	20	7,1	0,737
Ileus	16	7	2,2	9	3,9	0,303	7	2,6	9	3,2	0,802
Ischämie	13	7	2,2	6	2,6	0,780	5	1,9	8	2,9	0,578
Cholezystitis	18	11	3,4	7	3,1	1,000	8	3,0	10	3,6	0,812
Cholezystolithiasis	13	11	3,4	2	0,9	0,084	6	2,2	7	2,5	1,000
Karzinom	17	8	2,5	9	3,9	0,454	5	1,9	12	4,3	0,139
Sonstiger Tumor	14	8	2,5	6	2,6	1,000	6	2,2	8	2,9	0,789
Pneumonie	9	4	1,2	5	2,2	0,500	3	1,1	6	2,1	0,505
Sonstige^b^	330	201	62,6	129	56,6	0,158	166	62,2	163	58,2	0,382

^a^Mehrfachnennung bei einzelnen Patienten möglich

^b^Unter Sonstige zählen: u. a. Pankreatitis, periphere arterielle Verschlusskrankheit, Thrombose, Narbenbruch

Beim Konsilgrund war hinsichtlich des Geschlechts- und Altersunterschiedes kein klinisch bedeutsamer signifikanter Unterscheid festzustellen (Tab. 4).

**Table 4:** 

Konsilgrund^b^	Anzahl [*n*]	Geschlecht (m) [*n*]	Geschlecht (w) [*n*]	*p*-Wert	Altersgruppe (bis 65 Jahre) [*n*]	Altersgruppe (ab 66 Jahre) [*n*]	*p*-Wert
Mitbeurteilung	383	219	164	0,396	186	195	1,000
Therapieempfehlung	79	38	41	**0,049**	37	42	0,717
Verlaufskontrolle	88	53	35	0,725	48	40	0,247
Wiedervorstellung	13	9	4	0,572	5	8	0,578
Operationsindikation	134	81	53	0,615	67	67	0,766
Postoperative Wiedervorstellung	38	24	14	0,611	19	19	1,000

^a^Mehrfachnennung bei einzelnen Patienten möglich

^b^Reihenfolge nach Wertigkeit im klinischen Vorgehen

Signifikant häufiger erhielten die definitiven Diagnosen „akutes Abdomen“ (*p* *=* 0,000 und 0,000), „Abszess/Phlegmone“ (*p* *=* 0,001 und 0,033) und „Cholezystolithiasis“ (*p* *=* 0,020 und 0,002) eine Empfehlung zur elektiven Operation bzw. wurde eine unmittelbare Operationsindikation abgeleitet (Tab. 5) im Vergleich zu allen Fällen, bei denen eine Operation angezeigt war.

**Table 5:** 

Diagnosen (definitiv)			Operationsempfehlung (elektiv)* n* *=* 71			Operationsindikation *n* = 64		
		Anzahl gesamt	Anzahl [*n*]	Relativer Anteil [%]	*p*-Wert	Anzahl [*n*]	Relativer Anteil [%]	*p*-Wert
Wundheilungsstörung	Ja	39	3	7,7	0,345	1	2,6	**0,071**
	Nein	510	68	13,3		63	12,4	
Platzbauch	Ja	1	1	100,0	0,129	1	100,0	0,117
	Nein	548	70	12,8		63	11,5	
Unklares Abdomen	Ja	11	0	0,0	0,374	0	0,0	0,378
	Nein	538	71	13,2		64	11,9	
Akutes Abdomen	Ja	39	15	38,5	**0,000**	14	35,9	**0,000**
	Nein	510	56	11,0		50	9,8	
Hernie	Ja	20	5	25,0	0,162	0	0,0	0,151
	Nein	529	66	12,5		64	12,1	
Perforation	Ja	5	1	20,0	1,000	1	20,0	1,000
	Nein	544	70	12,9		63	11,6	
Abszess/Phlegmone	Ja	22	9	40,9	**0,001**	6	27,3	**0,033**
	Nein	527	62	11,8		58	11,0	
Hämatom	Ja	26	1	3,8	0,232	2	7,7	0,571
	Nein	523	70	13,4		62	11,9	
Blutung	Ja	26	4	15,4	0,762	4	15,4	0,756
	Nein	523	67	12,8		60	11,5	
Ileus	Ja	5	0	0,0	0,626	2	40,0	0,106
	Nein	544	71	13,1		62	11,4	
Ischämie	Ja	13	3	23,1	0,392	5	38,5	**0,011**
	Nein	536	68	12,7		59	11,0	
Cholezystitis	Ja	14	4	28,6	0,094	1	7,1	0,717
	Nein	535	67	12,5		63	11,8	
Cholezystolithiasis	Ja	9	4	44,4	**0,020**	5	55,6	**0,002**
	Nein	540	67	12,4		59	10,9	
Karzinom	Ja	8	2	25,0	0,605	1	12,5	1,000
	Nein	541	69	12,8		63	11,6	
Sonstiger Tumor	Ja	13	7	53,8	**0,000**	4	30,8	0,053
	Nein	536	64	11,9		60	11,2	
Pneumonie	Ja	10	1	10,0	1,000	1	10,0	–
	Nein	539	7	13,0		63	11,7	1,000
Sonstige^b^	Ja	309	31	10,0	**0,029**	39	12,6	0,503
	Nein	240	40	16,7		25	10,4	

^a^Mehrfachnennung bei einzelnen Patienten möglich

^b^Unter Sonstige zählen: u. a. Pankreatitis, periphere arterielle Verschlusskrankheit, Thrombose, Narbenbruch

Hierbei wurde bei *allen* diagnostizierten Fällen mit „Cholezystolithiasis“ (*n* *=* 9) eine notwendige Operation abgeleitet („Operationsempfehlung“ [*n* *=* *4*] oder „Operationsindikation“ [*n* *=* 5]).

## Diskussion

### Eigene Ergebnisse

Die chirurgische Konsiltätigkeit ist ein wichtiges Standbein für die suffiziente und vor allem zeitgerechte Klärung chirurgisch relevanter Fragestellungen fast in jeder medizinischen Einrichtung, so vor allem auch in einem Zentrum.

Die vorliegende klinikinterne chirurgische Studie stellt eine dringend notwendige systematische Aufarbeitung der chirurgischen Diensttätigkeit dar. Sie dient

- der chirurgischen Qualitätssicherung bei der klinischen Mitbetreuung von Patienten mit interdisziplinärem, hier chirurgischem Versorgungsbedarf in der täglichen allgemein- und viszeralchirurgischen Praxis im Rahmen der klinischen Versorgungsforschung,
- dem Klinikmarketing sowie Budgetsicherung durch Patientenaquise (und)
- nicht zuletzt der Versorgung von Notfällen.

Die Daten reflektieren anschaulich das tägliche Arbeitsprofil eines allgemein- und viszeralchirurgischen Oberarztes bei hohem Erfassungsgrad der aufgeführten Auswertparameter.

Limitierend sind:

- das unizentrische Studiendesign (sowie)
- die ausschließliche Erfassung eines einzelnen Akteurs (allgemein- und viszeralchirurgischer Oberarzt)

aufzuführen.

Zehn Jahre erlauben daneben durchaus mit der analysierten Fallzahl von ca. 550 ausgewerteten Patienten*innen die kompetente statistische Testung von Einflussfaktoren auf die chirurgische Entscheidungsfindung sowie die Ermittlung von Geschlechts- oder Altersunterschieden in Gegenüberstellung von Teilzeiträumen als auch einem elektiven und Notfallsetting.

Grundsätzlich galt es dabei zu klären, wie diese in Einklang mit den anderen Tätigkeiten während des chirurgischen Dienstes zu bekommen sind. Es gilt zu triagieren, inwieweit das gestellte Konsil dringend ist und ob ggf. eine Notfallsituation mit hochakutem Handlungsbedarf vorliegt, bei der eine Umstellung des Operationsplans erfolgen muss.

Vorherrschend bei den Anforderungen sind vornehmlich die Fächer Kardiologie, andere chirurgische Disziplinen und die Gastroenterologie. Dieses ist aber nicht verwunderlich, da diese Fächer häufig Erkrankungen behandeln, die chirurgische Intervention bzw. Nachbehandlung erfordern.

Es wurden im Verhältnis etwas mehr Frauen als Männer konsiliarisch vorgestellt.

Bei Betrachtung der Untergruppe des akuten Abdomens fiel auf, dass die Diagnose einer Darmischämie tendenziell etwas häufiger bei den weiblichen Patienten gestellt wurde, ein signifikanter Unterschied bestand nicht. An dieser Stelle soll nicht unerwähnt bleiben, dass es sich bei den festgestellten Ischämien hauptsächlich um Dünndarmischämien handelte. In geringerem Ausmaß waren auch Ischämien anderer Lokalisationen vertreten.

Bezüglich der festgestellten Erkrankungen in dieser Untergruppe gab es keine Auffälligkeiten. Häufig anzutreffen waren Ulkusperforationen, Cholezystitiden etc. Dieses deckt sich auch mit dem Literaturvergleich [1–5].

Bei der Auswertung fällt auf, dass ein relativ hohes Erkrankungsalter mit durchschnittlich 63 Jahren vorliegt. Der Anteil der über 65-Jährigen bei den gestellten Konsilen beträgt ca. 50 %, verbunden mit einem höheren Anteil an „Nebenerkrankungen“. Dieses zeigt eindrucksvoll die demografische Entwicklung der letzten Jahrzehnte hin zum höheren Lebensalter. Dieses gilt insbesondere auch für Sachsen-Anhalt, wo der Anteil an Rentner*innen überdurchschnittlich hoch mit 27 % zu finden ist. Im Vergleich liegt dieser in Gesamtdeutschland bei 22 % [6, 7]. In diesem Patientenkollektiv besteht häufig eine erhöhte Komorbidität [8]. Ein signifikanter Unterschied bei den gestellten Konsilen im Vergleich zu den Jüngeren unter 65 Jahren bestand aber nicht.

Die Krux ist, dass die bei allen Konsilen angegebene Verdachtsdiagnose nur zu 58 % mit der definitiven Diagnose übereingestimmt hat. Umgekehrt bedeutet dies, dass sich zu 42 % letztlich durch das kompetent durchgeführte allgemein-/viszeralchirurgische Konsil differente Diagnosen herausstellten.

Somit kann allein aus der Anforderung nicht interpretiert bzw. verlässlich abgeleitet werden, ob eine eilige oder sogar eine Notfallindikation besteht. An dieser Stelle sollte jedoch nicht unerwähnt bleiben, dass dieses nicht auf Inkompetenz der Konsilstellung hindeutet, da es sich aus analysierender chirurgischer Sicht um fachfremde Disziplinen handelt.

Eine Indikation zur Operation noch während des Dienstes wurde in 12 % der Fälle gestellt und trägt somit deutlich zur Dienstbelastung bei. Daraus ist zusätzlich ersichtlich, wie wichtig eine zeitnahe „Abarbeitung“ des Konsils ist. Somit sollte eine Beurteilung des Patienten unmittelbar bzw. schnellstmöglich nach Konsilstellung erfolgen. Deutlich wird auch, dass bei aller Priorität der körperlichen Untersuchung sowie der fachärztlichen Einschätzung im Zweifel eine erweiterte Diagnostik wie z. B. nicht selten durch ein CT erfolgen sollte, bei deren Indikationsstellung und Planung des Untersuchungsmodus der allgemein-/viszeralchirurgische Konsiliarius bereits schon – wie die klinische Praxis lehrt – einbezogen sein sollte, um eine kompetent ausgerichtete Untersuchung mit hoher Klärungsaussicht unter Nutzung und bestmöglicher Ausschöpfung des Potenzials der anberaumten Diagnostik zu gewährleisten.

Im untersuchten Kollektiv war dieses zu 40 % notwendig.

Wenn – der allgemein-/viszeralchirurgischen Konsilempfehlung folgend – die Patienten einer Operation zugeführt wurden, entsprachen die dort angetroffenen Krankheitsbilder in ihrem Diagnoseprofil der normalen Verteilung des akuten Abdomens (Cholezystitis, Ischämie etc.).

Ein weiterer Aspekt der konsiliarischen Tätigkeit ist monetärer Art. Auf diese Art und Weise können Patienten*innen zur operativen Therapie entweder notfallmäßig oder elektiv generiert werden. Bei insgesamt 24 % der vorgestellten Patienten schloss sich eine Operation entweder mit elektiver Aufnahme oder mit konsekutiver notfallmäßiger chirurgischer Übernahme an.

Limitierend an der Untersuchung ist, dass es sich um eine retrospektive Analyse prospektiv erhobener Daten einer einzelnen Person handelt.

Zudem wird eine Erhebung vorgelegt, die (lediglich) in einem einzelnen Zentrum durchgeführt wurde und damit nur mit Einschränkung allgemein übertragbar ist. Außerdem konnte keine detaillierte Aussage über die durchgeführten Untersuchungen im Rahmen der bildgebenden Diagnostik getätigt werden. Auch wenn im Normalfall bei den vorgefunden klinischen Fällen eine CT des Abdomens durchgeführt wurde.

Grundsätzlich wird jedoch das ganze Spektrum dieser nicht unwesentlich und wenig im Fokus stehenden Dienstaufgabe mit dem begründeten und systematischen Ansatz einer wissenschaftlichen Aufarbeitung widergespiegelt.

Besonders hervorzuheben ist hingegen, dass eindrucksvoll gezeigt wurde, dass die konsiliarische Tätigkeit einen nicht unerheblichen Anteil der Diensttätigkeit ausmacht.

Ferner wird durch die oben genannten Ergebnisse sehr deutlich, wie wichtig und teils entscheidend die eher zeitnahe und gewissenhafte „Abarbeitung“ der Konsile ist, da sich potenziell hinter jeder gestellten Konsilanfrage ein Notfall mit akutem Handlungsbedarf – gerade in chirurgischen Fachdisziplinen – verbergen kann.

Es konnte gezeigt werden, dass auch die Indikationsstellung und folgende effektive Planung elektiver Operationen elegant und vor allem mit flacher Hierarchie („kurze Dienstweg“) vorgenommen werden können und somit dazu beitragen, das Erlösaufkommen einer Klinik oder Abteilung zu entwickeln bzw. zu konsolidieren.

### Literaturvergleich

Direkte Vergleichskollektive in Bezug auf die Untersuchung des Konsiliarsystems zu finden, ist in der Literaturrecherche schwer möglich. Eventuell ist dies der Tatsache geschuldet, dass die in Deutschland anzutreffende Organisationsform nicht weltweit in der Form ausgeübt wird. Es werden somit vornehmlich Teilaspekte, wie z. B. die chirurgische Notfallversorgung des akuten Abdomens, untersucht.

Wenn man sich auf die epidemiologischen Daten und die Patienten, die einer Operation notfallmäßig zugeführt wurden, konzentriert, dann werden auch von anderen Forschungsgruppen ein erhöhtes Alter der untersuchten Patientenklientel mit ausgeprägter Komorbidität postuliert. Bei der Diagnostik gibt es einen Trend beim akuten Abdomen, insbesondere in unklaren Situationen, zu einer erweiterten Diagnostik mittels abdominal-CT-spezifischer Untersuchungsmodi. Hier wird nahezu einvernehmlich geraten, dass im Zweifel diese Untersuchung durchgeführt werden soll. Somit zeigt sich, wie in der vorliegenden Untersuchung auch festgestellt, ein Anstieg der vorgenommenen CT-Untersuchungen [9–12].

Aktuell wird in der Literatur ein besonderes Augenmerk auf die Verbesserung des zeitlichen Ablaufes bis zur Diagnosestellung gelegt. Es gibt immer mehr Kliniken, in denen die konsiliarische Beurteilung des akut zu behandelnden Patienten durch einen ausgebildeten „Notfallchirurgen“ durchgeführt wird. Die Daten mehrerer Gruppen deuten darauf hin, dass so einer möglichen Verschleppung des Diagnosezeitpunktes entgegengearbeitet werden kann und auf diese Weise die Patienten schneller einer erforderlichen Operation zugeführt werden können [13–18].

Zudem ist es hinweisend auf einen generell vorhandenen, nicht unbeträchtlichen Zeitaufwand, wenn extra Personal dafür bereitgestellt wird.

Für das Konsiliarsystem in Deutschland wäre dieses sicherlich nur partiell übertragbar, da wir bereits bei der Begutachtung ein fachärztliches Niveau haben. Zumal die dann gestellte definitive Diagnose sich zu 42 % von der in der Anforderung vermuteten unterscheidet. Dennoch sollte es den Anreiz geben, Patienten mit einer unklaren Diagnose, insbesondere wenn es sich um ein akutes Abdomen handelt, von dem erfahrensten chirurgischen Personal beurteilen bzw. begutachten zu lassen. Vielleicht ist es sogar sinnvoll, dass die Anforderungen zur Beurteilung eines akuten Abdomens getrennt von den restlichen Konsilen ggf. mit einem extra dafür bereitgestellten und dafür reservierten Telefon anzufordern sind.

## Fazit

Die Durchführung chirurgischer Konsile ist ein wichtiger Bestandteil der chirurgischen Dienstaufgabe. Da sich mit einem Anteil von ca. 12 % darüber Notfallindikationen für eine Operation ableiten und die im Konsil teilweise als harmlose Erkrankung dargestellte Verdachtsdiagnose zu 42 % falsch ist, sollten diese immer zeitnah durch eine persönliche oberärztliche Beurteilung abgearbeitet werden, um keine unnötige Gefährdung in Kauf zu nehmen.

Dieses unterstreicht nochmals, dass die Konsiltätigkeit verbunden mit den daraus folgenden Konsequenzen einer Operation einen erheblichen Zeitaufwand während des Dienstes darstellt. Zudem ist die Konsiliararbeit von monetärer Bedeutung, da über sie Patienten notallmäßig oder elektiv zu einem Anteil von 24 % zu einer operativen Therapie rekrutiert werden und somit nicht zuletzt besonders unter Berücksichtigung des Aspektes der Dienstleistung zu einem guten Klinikmarketing mit Steigerung des Ansehens der chirurgischen Kliniken beitragen (helfen).
